# Supplementary material for: BridgePRS leverages shared genetic effects across ancestries to increase polygenic risk score portability
Source: Nat Genet. 2023 Dec 20;56(1):180–6. doi: 10.1038/s41588-023-01583-9 (PMC10786716; doi:10.1038/s41588-023-01583-9)
Supplement: Supplementary file 1 — Supplementary Table 1 and Figs. 1–4. [file 41588_2023_1583_MOESM1_ESM.pdf]

nature genetics

Technical Report https://doi.org/10.1038/s41588-023-01583-9

BridgePRS leverages shared genetic effects across ancestries to increase polygenic risk score portability

In the format provided by the authors and unedited

	Mean R^2			
	Imputed variants		Genotyped variants	
	F-stat	P-value	F-stat	P-value
African	0.0413	0.0403	0.0413	0.0359
South Asian	0.0683	0.0688	0.0694	0.0646

Supplementary Table 1: Average R^2 results from *BridgePRS* ranking loci by the pseudo F-statistic versus P-value from the European GWASs across the 19 traits analysed in this paper in African and South Asian UKB samples. Results are shown for analyses using both imputed and genotyped variants. All other results presented in the paper used the pseudo F-statistic loci ranking.

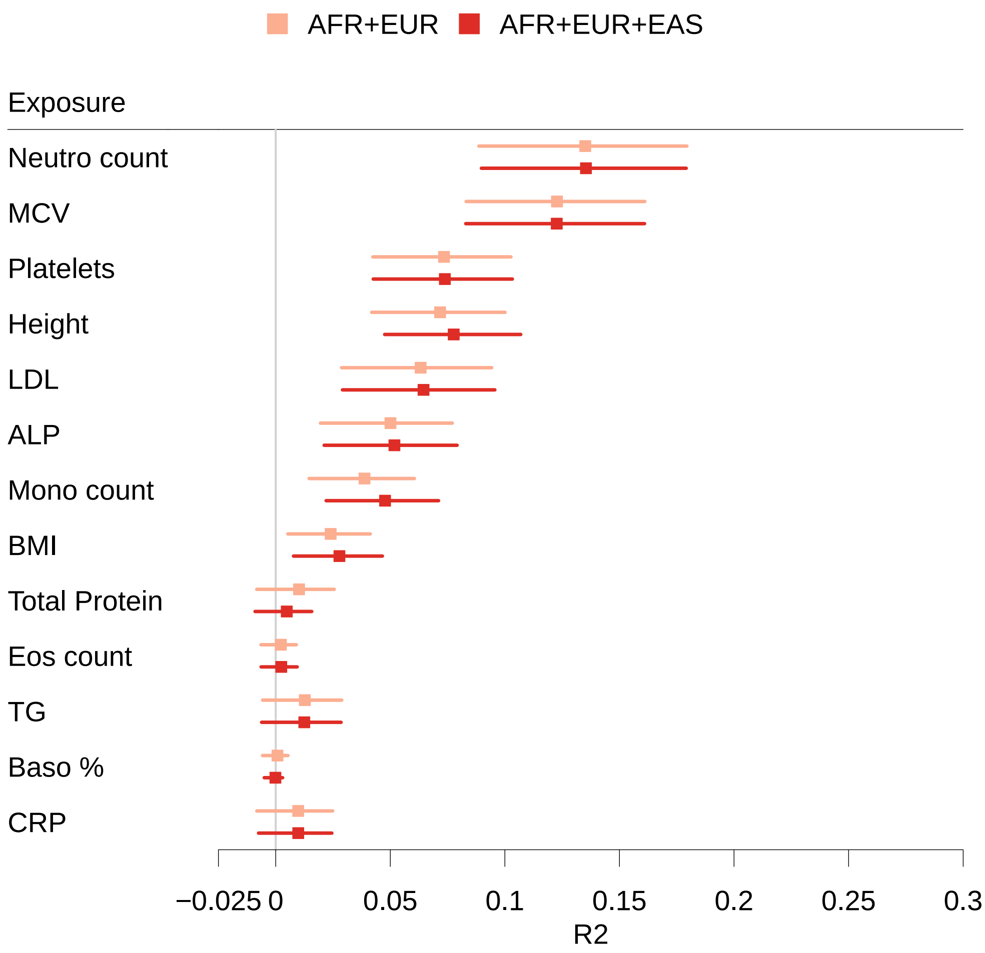

Supplementary Figure 1: Comparison of phenotypic variance explained (R^2 point estimates and 95% confidence intervals) by *BridgePRS* in samples of African ancestry in the UK Biobank training PRS using (1) AFR+EUR UKB summaray statistic data and (2) AFR+EUR UKB +EAS BBJ summaray statistic data.

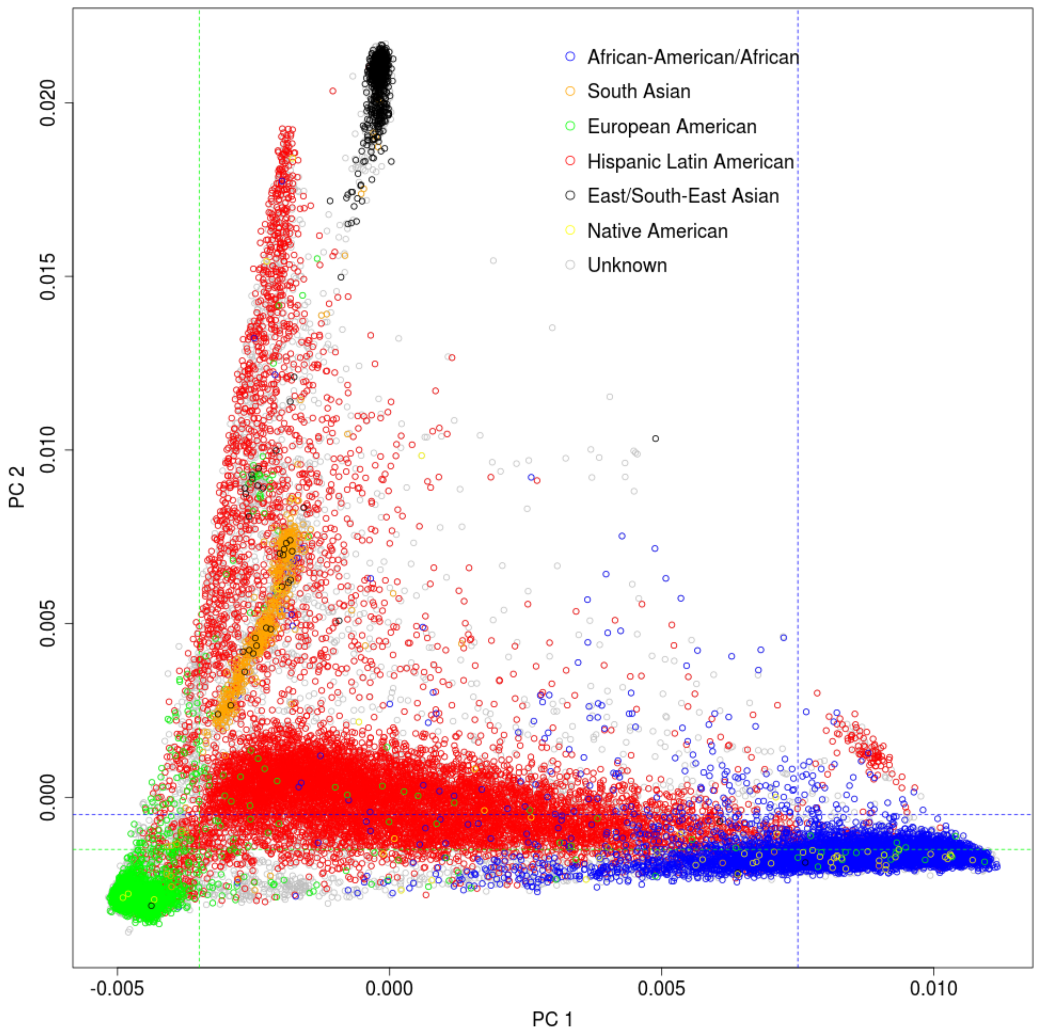

Supplementary Figure 2: Plot of PC 1 v PC 2 for Bio*Me* samples. Samples are coloured by self reported ancestry. Samples in bottom right corner indicated by blue dashed line were defined as African ancestry in our analyses.

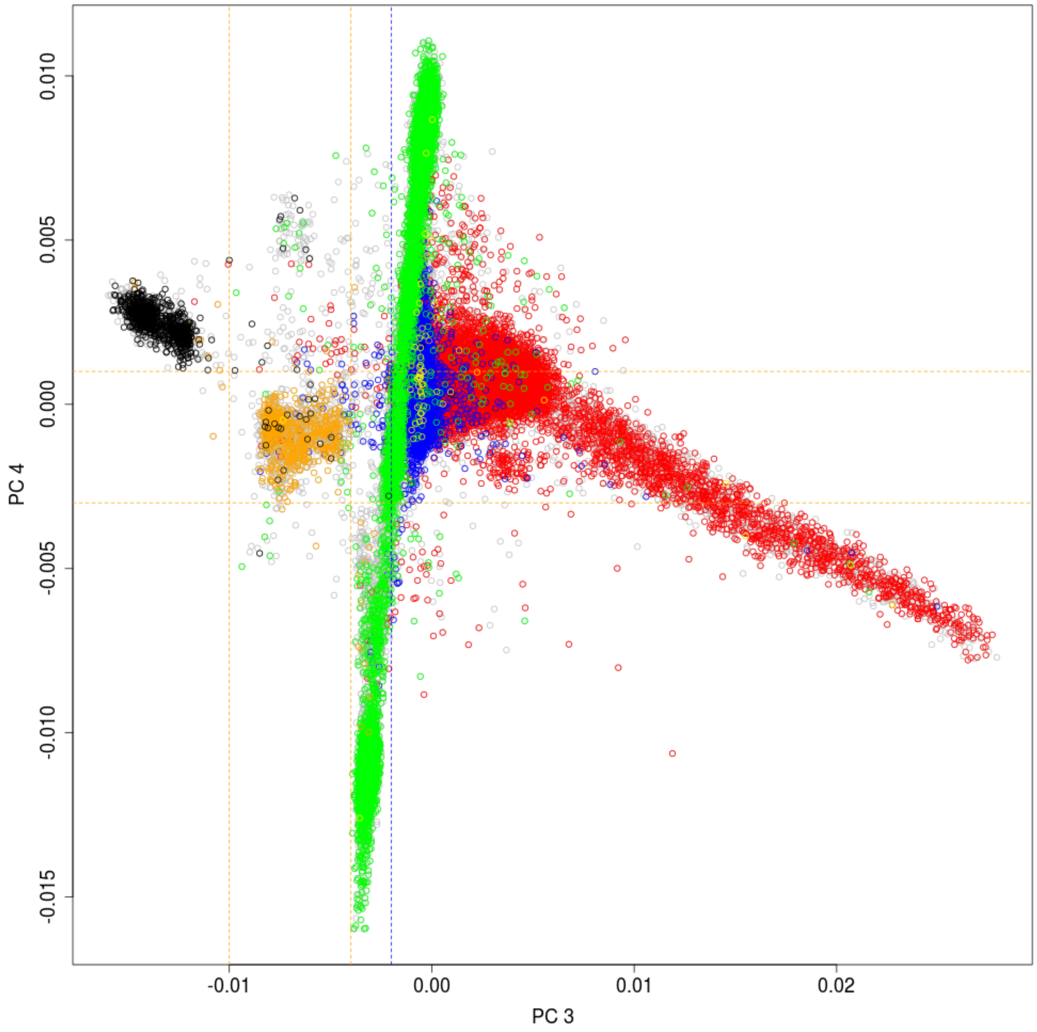

Supplementary Figure 3: Plot of PC 3 v PC 4 for Bio*Me* samples. Samples are coloured by self reported ancestry. Samples in box defined by orange lines were defined as South Asian ancestry in our analyses. Samples to left of blue vertical were excluded from African analyses.

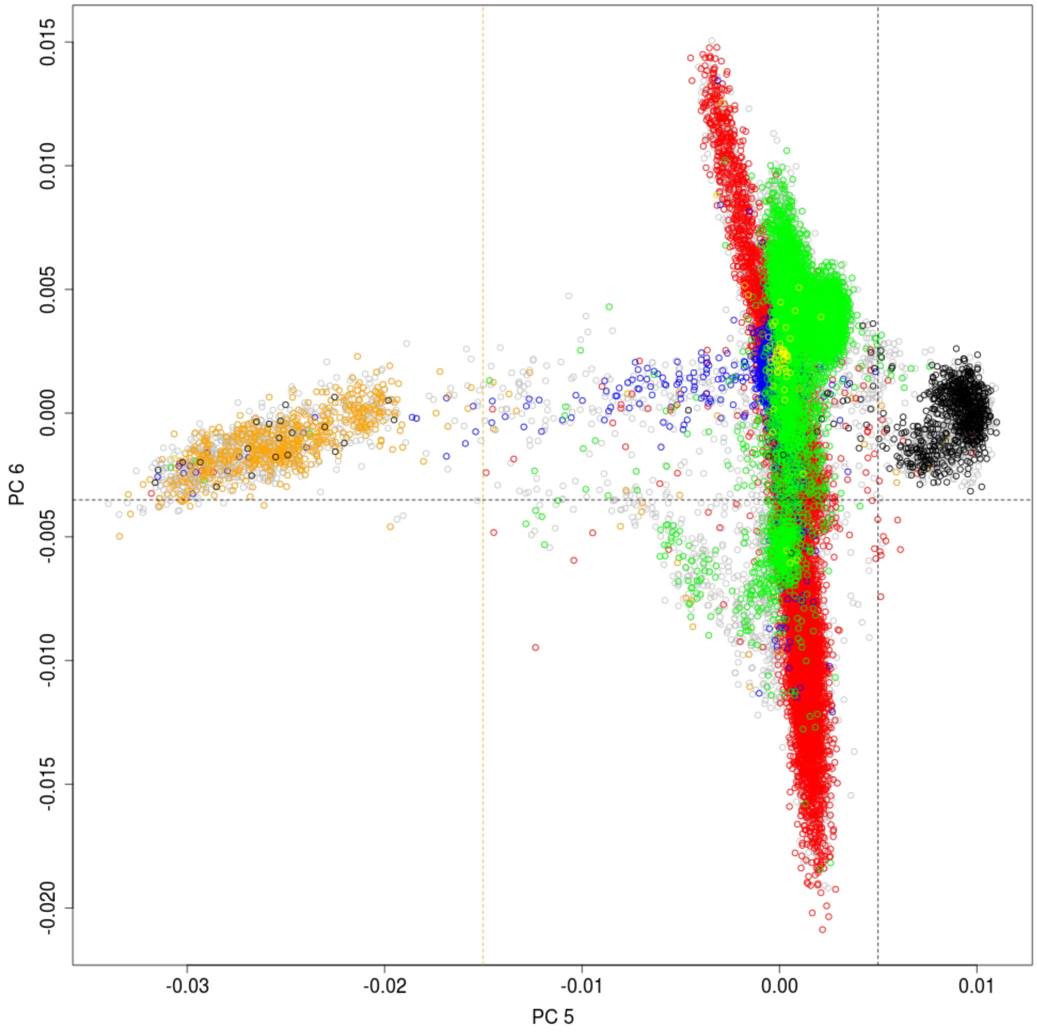

Supplementary Figure 4: Plot of PC 5 v PC 6 for Bio*Me* samples. Samples are coloured by self reported ancestry. Samples to right of orange vertical were excluded from South Asian analyses.
