# Supplementary material for: BridgePRS leverages shared genetic effects across ancestries to increase polygenic risk score portability
Source: Nat Genet. 2023 Dec 20;56(1):180–6. doi: 10.1038/s41588-023-01583-9 (PMC10786716; doi:10.1038/s41588-023-01583-9)
Supplement: Supplementary file 2 — Reporting Summary [file 41588_2023_1583_MOESM2_ESM.pdf]

nature portfolio

Corresponding author(s): Clive Hoggart, Paul F. O'Reilly

Last updated by author(s): Jun 24, 2023

Reporting Summary

Nature Portfolio wishes to improve the reproducibility of the work that we publish. This form provides structure for consistency and transparency in reporting. For further information on Nature Portfolio policies, see our Editorial Policies and the Editorial Policy Checklist.

Statistics

For all statistical analyses, confirm that the following items are present in the figure legend, table legend, main text, or Methods section.

n/a	Confirmed	
☐	☒	The exact sample size (*n*) for each experimental group/condition, given as a discrete number and unit of measurement
☐	☒	A statement on whether measurements were taken from distinct samples or whether the same sample was measured repeatedly
☐	☒	The statistical test(s) used AND whether they are one- or two-sided<br>*Only common tests should be described solely by name; describe more complex techniques in the Methods section.*
☐	☒	A description of all covariates tested
☒	☐	A description of any assumptions or corrections, such as tests of normality and adjustment for multiple comparisons
☐	☒	A full description of the statistical parameters including central tendency (e.g. means) or other basic estimates (e.g. regression coefficient) AND variation (e.g. standard deviation) or associated estimates of uncertainty (e.g. confidence intervals)
☐	☒	For null hypothesis testing, the test statistic (e.g. *F*, *t*, *r*) with confidence intervals, effect sizes, degrees of freedom and *P* value noted<br>*Give P values as exact values whenever suitable.*
☐	☒	For Bayesian analysis, information on the choice of priors and Markov chain Monte Carlo settings
☒	☐	For hierarchical and complex designs, identification of the appropriate level for tests and full reporting of outcomes
☐	☒	Estimates of effect sizes (e.g. Cohen's *d*, Pearson's *r*), indicating how they were calculated

Our web collection on statistics for biologists contains articles on many of the points above.

Software and code

Policy information about availability of computer code

Data collection

Provide a description of all commercial, open source and custom code used to collect the data in this study, specifying the version used OR state that no software was used.

Data analysis

BridgePRS software, example data and tutorial for guiding its use are available from www.bridgeprs.net. Source code, to which www.bridgeprs.net links, is available from https://github.com/clivehoggart/BridgePRS, DOI badge https://zenodo.org/badge/latestdoi/452809505. Scripts used for all analyses are available on GitHub: https://github.com/clivehoggart/BridgePRS data
All other code
- PLINK v1.90: https://www.cog-genomics.org/plink/1.9/
- PLINK v2: https://www.cog-genomics.org/plink/2.0/
- LDSC version 1.01: https://github.com/bulik/ldsc
- METAL version 2011-03-25 - http://csg.sph.umich.edu/abecasis/metal/
- IMPUTE2 v2 https://mathgen.stats.ox.ac.uk/impute/impute_v2.html
- R version 4.0.3 https://cran.r-project.org
- Ridge regression - glmnet package (version 4.0-2) https://cran.r-project.org/web/packages/glmnet/index.html
- bootstrapping - boot package (version 1.3.25) https://cran.r-project.org/web/packages/boot/index.html
- PRS-CSx v1.0.0 (https://github.com/getian107/PRScsx)
- PRS-CS v1.0.0 (https://github.com/getian107/PRScs)
- PRSice-2 v2 (https://www.prsice.info}
- HAPGEN v2.2.0 (01/04/2011): https://mathgen.stats.ox.ac.uk/genetics_software/hapgen/hapgen2.htm

For manuscripts utilizing custom algorithms or software that are central to the research but not yet described in published literature, software must be made available to editors and reviewers. We strongly encourage code deposition in a community repository (e.g. GitHub). See the Nature Portfolio guidelines for submitting code & software for further information.

Data

Policy information about availability of data

All manuscripts must include a data availability statement. This statement should provide the following information, where applicable:

- Accession codes, unique identifiers, or web links for publicly available datasets
- A description of any restrictions on data availability
- For clinical datasets or third party data, please ensure that the statement adheres to our policy

Publicly available data used to generate the simulated data are available from the following sites: 1000G Phase 3 reference panels: https://mathgen.stats.ox.ac.uk/impute/1000GP Phase3. html and genetic maps for each subpopulation: ftp.1000genomes.ebi.ac.uk/vol1/ ftp/technical/working/20130507 omni recombination rates
UK Biobank genotype and phenotype data were obtained from the UK Biobank Resource under applica- tion 18177 https://www.ukbiobank.ac.uk/enable-your-research/approved-research/multi-trait-gwas-analyses-in-the-uk-biobank. UK Biobank Quality Control information (missingness, allele frequency, Hardy Weinberg Equilibrium) was obtained from UK Biobank resource 531: https://biobank.ctsu.ox.ac.uk/crystal/refer.cgi?id=531
Recruitment and enrollment of participants into the Mount Sinai BioMe Biobank is IRB and HIPAA approved. It is an electronic medical record-linked biobank which allows the use of de-identified samples linkable to past, present and future clinical information from electronic health records at Mount Sinai. Biome contains protected health information and is thus under controlled access. Application to access the data can be made to biome@mountsinai.org, also see https://icahn.mssm.edu/research/ipm/programs/biome-biobank.
BBJ summary statistics were downloaded from PheWeb: https://pheweb.jp.
SNP weights for the polygenic risk scores estimated by BridgePRS in this paper are available on Github https://github.com/clivehoggart/BridgePRS data

Field-specific reporting

Please select the one below that is the best fit for your research. If you are not sure, read the appropriate sections before making your selection.

☒ Life sciences ☐ Behavioural & social sciences ☐ Ecological, evolutionary & environmental sciences

For a reference copy of the document with all sections, see nature.com/documents/nr-reporting-summary-flat.pdf

Life sciences study design

All studies must disclose on these points even when the disclosure is negative.

Sample size	Biobank analyses: We used all samples that were available for trait analyses in Biobanks, sample sizes for each phenotype in each ancestral population in UKB, BBJ and Biome are reported in Supplementary Tables 1 and 2.<br><br>Simulation analyses: Sample sizes for simulation studies were chosen as those typical for current GWASs: 80K European, 20K non-European. We show in the manuscript that there is a linear relationship between GWAS sample size and heritability, therefore, we performed simulation at 3 levels of heritability: 25%, 50% and 75%, the later two heritabilities are equivalent to doubling and trebling the sample size respectively with a heritability of 25%. To confirm the relationship between heritability and sample size analyses were run with half the sample size: 40K European, 10K non-European.
Data exclusions	Standard genotype quality controls were performed in both cohorts and are reported in the Methods. In UKB, samples with phenotypic values 6 standard deviation away from the mean were excluded. In Biome samples with phenotypic values 3 standard deviation away from the mean were excluded
Replication	Biobank analyses: PRS estimated using UKB and BBJ summary data were replicated in (1) unseen UKB samples and (2) the independent Mount Sinai Biome Biobank. Confidence intervals (CIs) for the R2 in the replication cohorts were calculated via boot strapping of 10,000 replicates, resulting CIs were symmetrically distributed around the mean values indicating consistency.<br>Simulation analyses: Each simulation setting was repeated 10 times. Results were consistent across replicates.
Randomization	UKB samples were randomly assigned to training, test and validation cohorts In each ancestral population.
Blinding	Researchers were blinded to group assignments. All phenotypes analyses were continuous, therefore phenotype blinding is not applicable.

Reporting for specific materials, systems and methods

We require information from authors about some types of materials, experimental systems and methods used in many studies. Here, indicate whether each material, system or method listed is relevant to your study. If you are not sure if a list item applies to your research, read the appropriate section before selecting a response.

Materials & experimental systems

n/a	Involved in the study
☒	☐ Antibodies
☒	☐ Eukaryotic cell lines
☒	☐ Palaeontology and archaeology
☒	☐ Animals and other organisms
☐	☒ Human research participants
☒	☐ Clinical data
☒	☐ Dual use research of concern

Methods

n/a	Involved in the study
☒	☐ ChIP-seq
☒	☐ Flow cytometry
☒	☐ MRI-based neuroimaging

Human research participants

Policy information about studies involving human research participants

Population characteristics	The UK Biobank is a population cohort. The Mount Sinai BioMe Biobank is a ~60k patient electronic medical record-linked biobank at Mount Sinai hospital and enables researchers to rapidly and efficiently conduct genetic, epidemiologic, molecular, and genomic studies on large collections of research specimens linked with medical information.<br>It has been enrolling since September 2007.<br>The ~60k participants represent diverse ancestry classified as African American ~20%, European American ~29%, East Asian (~4%) , South Asian (~3%), Hispanic (~36%) and OTHER (~9%) group
Recruitment	This research has been conducted using the UK Biobank Resource under Application Number 18177 (P.F.O'Reilly). The Mount Sinai Biome Biobank recruited patients visiting Mount Sinai hospital.
Ethics oversight	The study protocols were approved by the institutional review board at the Icahn School of Medicine at Mount Sinai. Participants from the UK Biobank provided written informed consent (Information available at https://www.ukbiobank.ac.uk/2018/02/gdpr/). All DNA samples and data in this study were pseudonymized.

Note that full information on the approval of the study protocol must also be provided in the manuscript.
